# Identification of *Aedes aegypti* and *Aedes albopictus* eggs based on image processing and elliptic fourier analysis

**DOI:** 10.1038/s41598-023-28510-6

**Published:** 2023-10-13

**Authors:** Nikko Prayudi Gunara, Endra Joelianto, Intan Ahmad

**Affiliations:** 1 Instrumentation and Control Master Program, Institut Teknologi Bandung, Bandung, 40132 Indonesia; 2 Instrumentation and Control Research Group, Institut Teknologi Bandung, Bandung, 40132 Indonesia; 3 Biological Resource Management Research Group, Institut Teknologi Bandung, Bandung, 40132 Indonesia

**Keywords:** Biological techniques, Engineering, Mathematics and computing

## Abstract

Dengue hemorrhagic fever is a worldwide epidemic caused by dengue virus and spread by infected female mosquitoes. The two main mosquito species vectors of the dengue virus are *Aedes aegypti* and *Aedes albopictus*. Conventionally, the identification of these two species’ egg is time-consuming which makes vector control more difficult. However, although attempts on efficiency improvements by providing automatic identification have been conducted, the earliest stage is at the larval stage. In addition, there are currently no studies on classifying to distinguish the two vectors during the egg stage based on their digital image. A total of 140 egg images of *Aedes aegypti* and *Aedes albopictus* were collected and validated by rearing them individually to become adult mosquitoes. Image processing and elliptic Fourier analysis were carried out to extract and describe the shape difference of the two vectors’ eggs. Machine learning algorithms were then used to classify the shape signatures. Morphometrically, the two species’ eggs were significantly different, which *Aedes albopictus* were smaller in size. Egg-shape contour reconstructions of principal components and Multivariate Analysis of Variance (MANOVA) revealed that there is a significant difference (*p* value $< 0.000$) in shape between two species’ eggs at the posterior end. Based on *Wilk’s lambda* of the MANOVA results, the classification could be done using only the first 3 principal components. Classification of the test data yielded an accuracy of 85.00% and F1 score 84.21% with Linear Discriminant Analysis applying default hyperparameter. Alternatively, *k*-Nearest Neighbors with optimal hyperparameter yielded a higher classification result with 87.50% and 87.18% of accuracy and F1 score, respectively. These results demonstrate that the proposed method can be used to classify *Aedes aegypti* and *Aedes albopictus* eggs based on their digital image. This method provides a foundation for improving the identification and surveillance of the two vectors and decision making in developing vector control strategies.

## Introduction

Dengue Hemorrhagic Fever (DHF) is a dangerous disease and has received a lot of attention in the world for decades, especially in tropical and sub-tropical countries. DHF is a disease caused by the dengue virus and its spread is caused by infected female mosquitoes. The two main mosquito species of dengue vector virus that cause dengue fever are *Aedes aegypti* and *Aedes albopictus*^1, 2^. Based on a modelling study, it is estimated that there are 390 million cases of the disease caused by the dengue virus annually from all over the world^3^. Due to the limitations and restricted use of available effective dengue vaccines, dengue virus vector control becomes the main choice in the efforts to prevent and to reduce the spread of DHF^2, 4^.

An attempt to detect the presence of the dengue virus vector that has been widely used is by using ovitraps. Several studies show ovitraps are able to detect the eggs, larvae, pupae^5^, and adult *Aedes aegypti* mosquitoes^6^. An ovitrap-based surveillance is usually done by installing a number of ovitraps in a monitoring area. Then identification and analysis are carried out at the mature and immature stages to estimate the abundance of a particular mosquito species in each area, although the monitoring systems are complex and changeable^7–12^. Based on the results of the analysis, further control strategies can be designed.

*Ae. aegypti* and *Ae. albopictus* are mosquitoes that lay eggs in artificial and natural water reservoirs, such as old tires, plastic, and tree holes. After the eggs hatch, the larvae will develop for several days to weeks depending on the temperature until they become pupae. Pupae will develop for 2–3 days until they become adult mosquitoes. Female *Aedes* mosquitoes can produce 100–200 eggs after receiving blood meal^1^. They lay their eggs on a moist surface at varying distances from the surface of the water. In the mature stage, *Ae. aegypti* and *Ae. albopictus* can be distinguished by observing the body parts of the mosquito. The easiest and quite clear observations to distinguish the two types of mosquitoes are based on the thorax part of the mosquito. Thorax mosquito *Ae. aegypti* has a pattern of two thin horizontal white lines in the middle and is surrounded by a curved line in the shape of a lute. While the thorax of *Ae. albopictus* has a pattern of one wide horizontal white line in the middle^1^. However, in the immature stage, identification of the eggs is still a challenge in ovitraps-based surveillance. Conventionally, the scanning electron microscopy (SEM) has been commonly used to study and identify the parameters to distinguish *Aedes* eggs^13–15^, specifically *Ae. aegypti* and *Ae. albopictus* eggs^16–18^. SEM has advantages in presenting a high resolution and detailed observation of fine structure of the eggs.

One of the earliest studies of *Aedes* eggs using SEM was presented in^13^ and^14^. In these studies, the surface structures were identified to distinguish the *Aedes* eggs including *Ae. aegypti* and *Ae. albopictus*. The authors reported key characteristics based on the pattern of reticulation, shape, and dimensions of papillae. However, in these papers, *Ae. aegypti* and *Ae. albopictus* were found similar with the same key characteristics. In^16^, the first comparison of *Ae. aegypti* and *Ae. albopictus* eggs was reported. In this paper, the micropylar collar was found to differentiate the eggs. In terms of shape, *Ae. albopictus* was known to be more tapered at the posterior end. The paper that more specific in studying the differentiation of *Ae. aegypti* and *Ae. albopictus* eggs is presented in^17^. The authors reported among 33 attributes which includes egg dimensions, micropylar apparatus, and outer chorionic cells dimension and density, these species’ eggs were 48,48% significantly different. In terms of shape, both species’ eggs were found to be cigar-shaped and tapered at ends. However, *Ae. albopictus* eggs are more tapered posteriorly. In^18^, an observation of the *Aedes* eggs using SEM with detailed color images was presented. The authors reported that these species’ eggs were similar and could be distinguished by their micropylar collar and central tubercles size. In terms of shape, *Ae. albopictus* is known to be strongly tapered from the widest point to the end of the egg. The latest study of *Ae. aegypti* and *Ae. albopictus* eggs differentiation was presented in^19^. In this paper, X-ray computed microtomography (micro-CT) was used. The proposed technique helped to measure the parameters directly, including length, surface area, volume, and eccentricity. In terms of shape, *Ae. albopictus* were found to be more tapered at ends presented by smaller eccentricity values.

The studies of *Ae. aegypti* and *Ae. albopictus* eggs using SEM and micro-CT yielded visible attributes to differentiate these species’ eggs for identification. However, these methods are time-consuming due to the transportation problems when the samples are far from the central laboratory^20^, stressfulness for the experts who manually identify each sample^21^, and complicated procedures. With consideration of the short life span of mosquitoes, these conventional methods make vector control more difficult. This problem motivates the need for automatic identification with minimal human expert intervention.

Although several attempts based on technology solutions have been made to improve the efficiency in vector surveillance^20–24^, the earliest stage that can be done for automatic identification is at the larval stage. Current attempts on mosquito egg identification^25–28^ still focus on counting the number of *Ae. aegypti* and *Ae. albopictus* eggs in ovitraps without identification^29^. By being able to identify at the egg stage, a more efficient identification process can be produced.

Recently, Elliptic Fourier Analysis (EFA) have been widely used in extracting and describing the shapes of agriculture products^30–33^, vectors’ egg^34^ and body morphology^35^. Based on previously described studies, it was stated that the *Ae. albopictus* eggs are more tapered at the posterior end^16–18^. Thus, we hypothesize that they could be classified by their shape. In this paper, the classification is carried out using a Linear Discriminant Analysis^30, 31, 35, 36^ and several alternative classification algorithms.

The objectives of this paper are to propose automatic identification of *Ae. aegypti* and *Ae. albopictus* eggs based on EFA and machine learning algorithms. The proposed method also uses a low-cost system based on a cellphone camera and a macro lens extension to provide easier implementation in a real case surveillance.

## Material and methods

### Data collection

The egg samples used in this paper were *Ae. aegypti* and *Ae. albopictus* eggs. *Ae. aegypti* eggs were obtained from the PPR Laboratory Research Station of the School of Life Sciences and Technology, Institut Teknologi Bandung, Indonesia ($6^{\circ }51'\,26.0''\,\hbox {S},\, 107^{\circ }37'\,29.9''\,\hbox {E}$). Meanwhile, *Ae. albopictus* eggs were obtained by ovitraps-based collection. The ovitraps were placed at two outdoor parks, namely ITB’s Botani Park ($6^{\circ }53'25.4'' \,\hbox {S},\, 107^{\circ }36'\,30.8''\,\hbox {E}$) and Ganesha Park ($^{\circ }53'\,38.1''\,\hbox {S},\, 107^{\circ }36'\,37.6''\,\hbox {E}$). Both parks are located in Bandung, Indonesia. Egg samples of *Ae. aegypti* and *Ae. albopictus* were dried at room temperature ($\pm 26\, ^{\circ }\hbox {C}$) and stored in a ziplock bag. Egg samples from both mosquito species were not older than 2 months to prevent deformation. The eggs were validated by individual rearing process until they become adult mosquitoes. When the mosquito had become an adult, the validation was done by observing the thorax of the mosquito. From 128 samples each of species’ egg and 16 days of rearing process, 68 and 74 samples of *Ae. aegypti* and *Ae. albopictus* had become adult mosquitoes. This process yielded validated samples of 68 *Ae. aegypti* (100%), 72 *Ae. albopictus* (97.3%) and 2 unidentified mosquitoes (2.7%).

**Figure 1:**
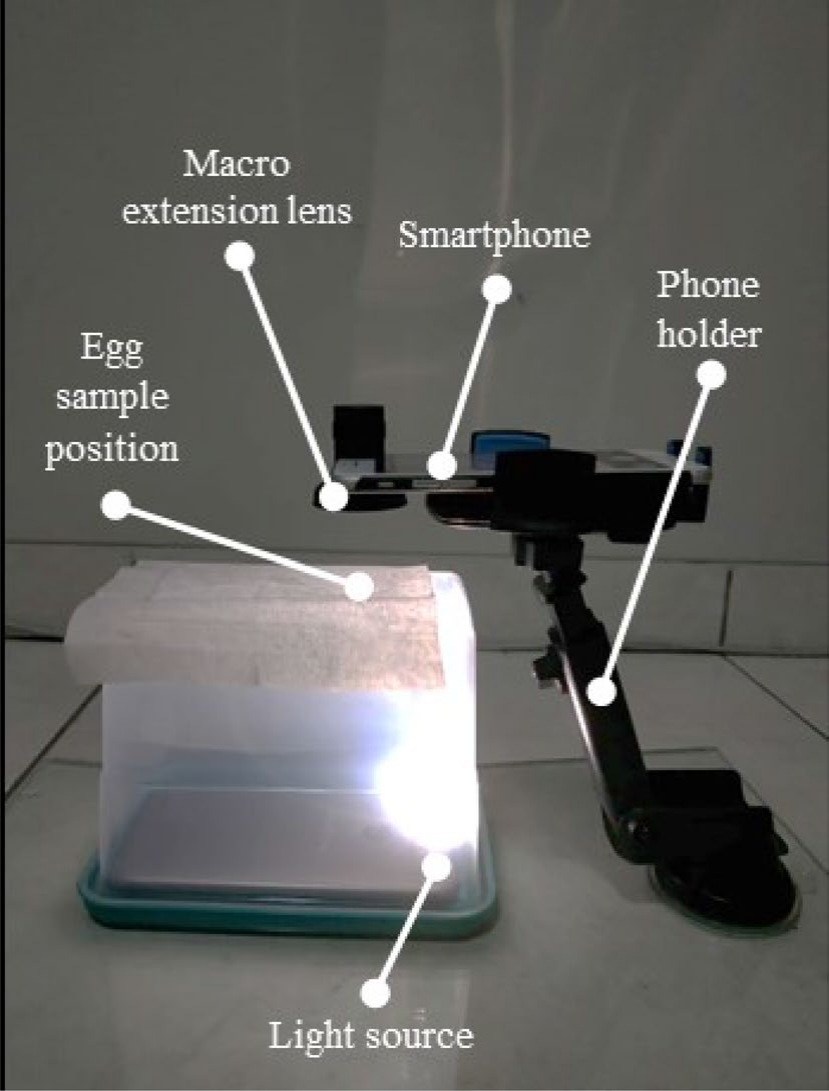
Low-cost image acquisition system design.

**Figure 2:**
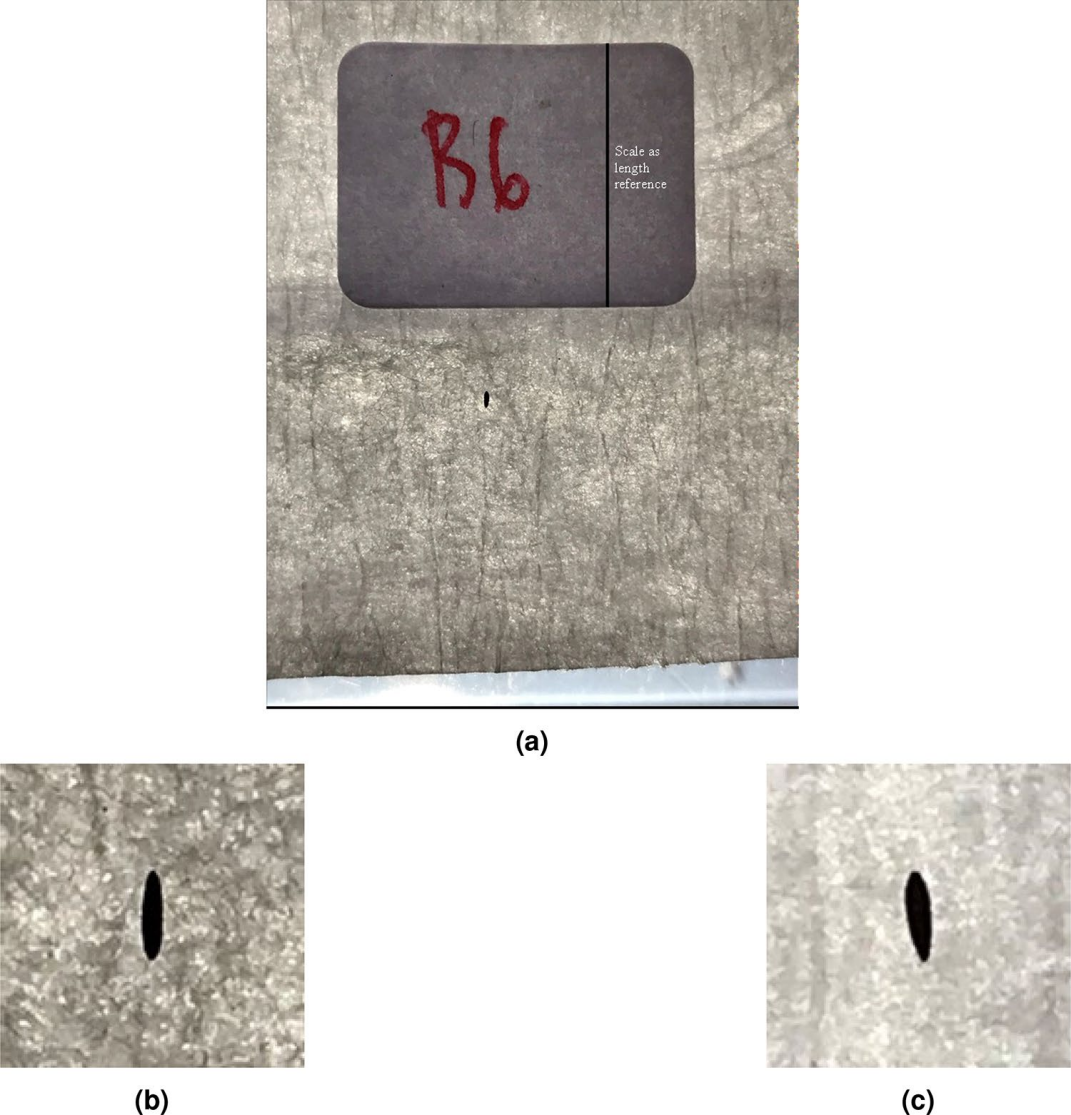
(**a**) Original, (**b**) cropped image of *Ae. aegypti*, and (**c**) *Ae. albopictus*.

A smartphone camera-based image data acquisition system was designed to collect image data. The smartphone used to capture images was *iPhone 7* with camera resolution of up to 12 MP and a focal length of 28 mm. To bring the camera’s focal point closer, an *Apexel APL-HD5M* macro lens extension was used. With the extension of the macro lens, the minimum focus distance from the phone camera became $\pm 4\,\hbox {cm}$, and 10 times magnification was obtained. The phone was placed on a phone holder and positioned facing perpendicularly to the base. The eggs were then individually placed at the distance of the closest focal point just below the camera on a transparent surface with filter paper and the image data was taken. Images data was taken with a digital zoom of 2.5 times. Furthermore, a light source was given from below as in^32, 33^. This was done to avoid shadows from the egg and, therefore, clear individual egg shape was obtained. Overall, the image acquisition system is shown in Fig. 1. The egg image data set are then manually cropped so that it is in a square form and the eggs are positioned with a uniform orientation with the posterior end is at the top of the image. An example of a cropped egg image data sample is shown in Fig. 2b,c.

### Image processing

**Figure 3:**
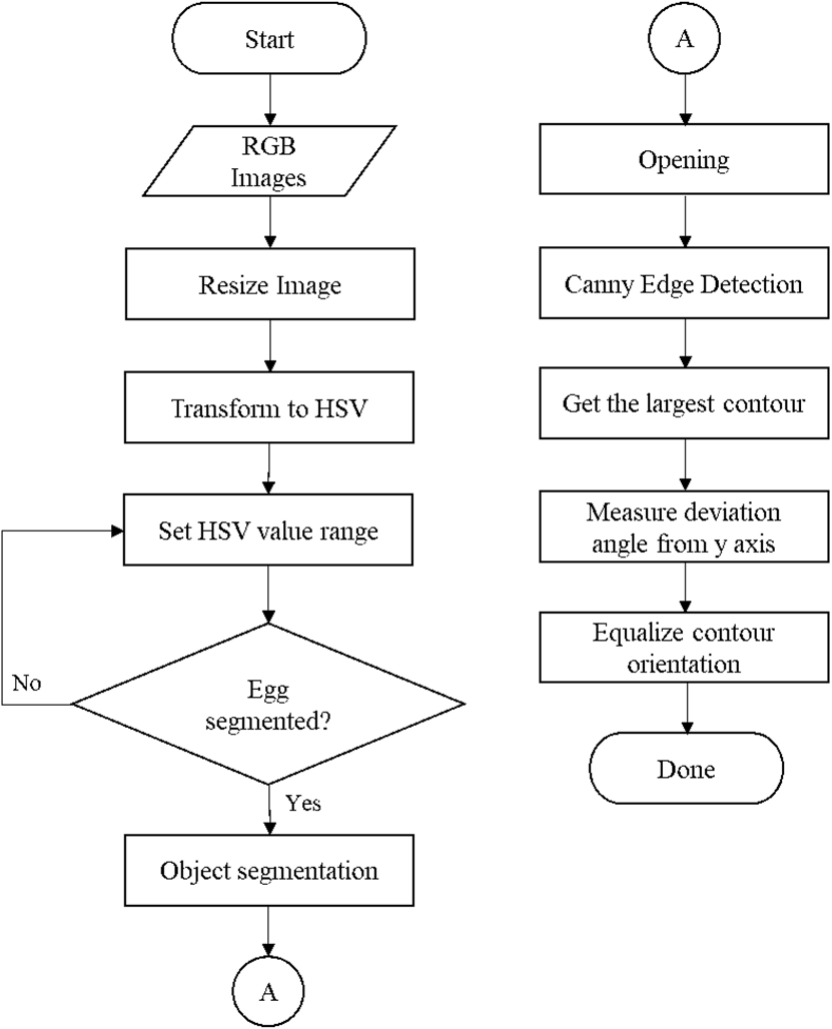
Image processing flowchart.

**Figure 4:**
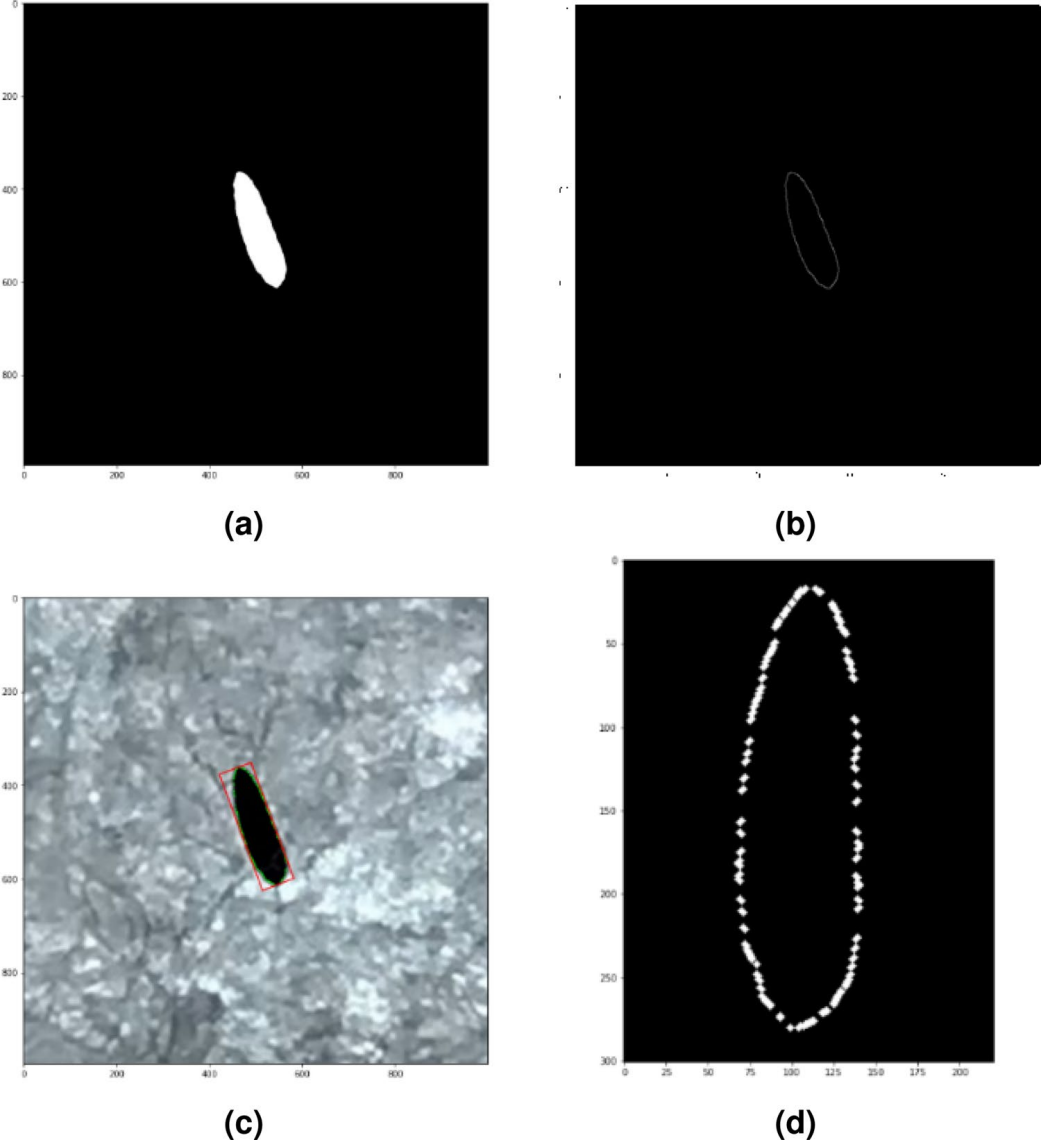
(**a**) Binary image of segmentation process, (**b**) Canny edge detection result, (**c**) measured orientation deviation, (**d**) corrected orientation contour.

**Figure 5:**
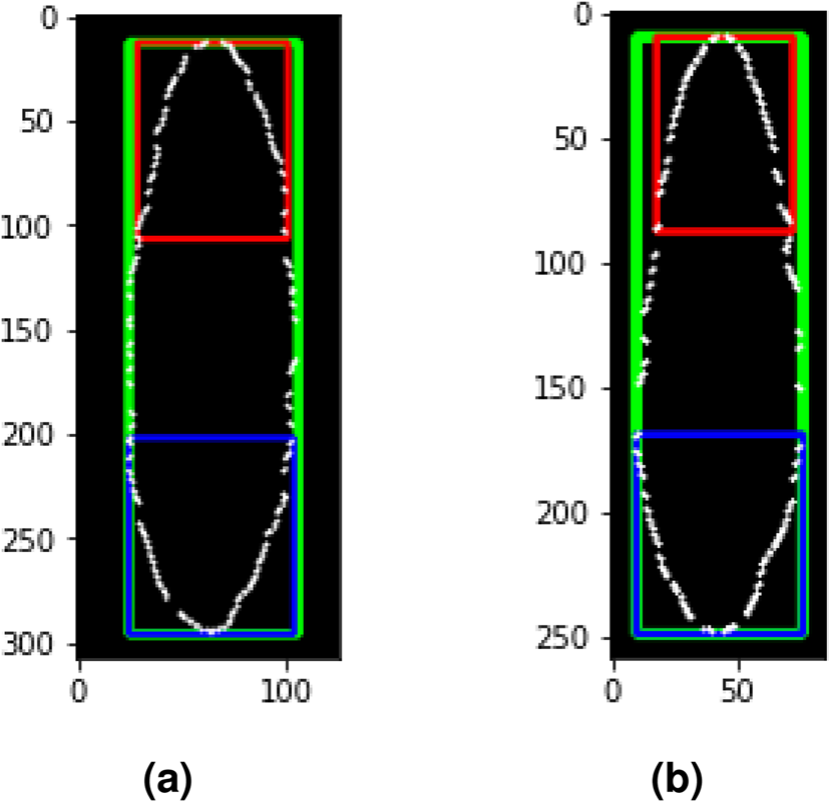
(**a**) Egg’s dimensions measurement of *Ae. aegypti* and (**b**) *Ae. albopictus*. Green box measures length and width of the egg. Red box measures width at 1/3 posterior end. Blue box measures width at 1/3 anterior end.

This section presents  the image processing method to obtain the contours of the collected egg images. This image processing began with resizing the resolution of the image up to 5 times of its initial size. This was done to make the eggs contours become smoother and more appropriate. The resizing process was done by up-sampling the image resolution with an interpolation method. Next, segmentation in the HSV color space was carried out. When the segmented binary image had been obtained, the morphological opening operation was performed to remove small pixels outside the egg object and to smooth the contours of the segmented egg's silhouette. An example of a binary image of this segmentation process is shown in Fig. 4a. Then to get information about the shape of the egg from the binary image, the *Canny* edge detection^37^ was used. Based on the resulting edge outline as shown in Fig. 5b, the egg contours could be extracted. Although every egg object in the image had been positioned uniformly, some eggs do not yet have the same orientation angle as shown in Fig. 4c. Therefore, it is necessary to measure the deviation of the egg contour and correct them. The overall steps performed in this image processing are described in the flowchart in Fig. 3.

This image processing was done using the *OpenCV* library^38^ and programmed in *Python 3.7.3*. The result of this stage was the egg contour points with uniform orientation as shown in Fig. 4d. This was done to ensure that each egg contour can be compared and then to measure the dimensions of the eggs.

### Egg’s dimensions measurement

The measurement of egg dimensions was carried out based on the egg's contour points. From the contour points, the dimensions of the egg in the image can be measured in pixels. To convert the dimensions from pixels to actual units, a length scale of known size was used. The scale used was the length of the label paper in the image data before the cropping process as shown in Fig. 2a. A total of 20 label papers were measured using a vernier caliper with an accuracy of 0.05 mm and an average reference length of 8.97 mm was obtained. Based on this value, the length of a pixel in millimeters can be calculated using the ratio of the reference length in millimeters to the reference length in pixels defined by following,

1 $$\begin{aligned} PL_{mm} = \frac{RL_{mm}}{RL_{pixel}} \end{aligned}$$

where $RL_{mm}$ is the reference length in millimeters and $RL_{pixel}$ is the reference length in pixels. By multiplying the $PL_{mm}$ value with the egg dimensions measured in pixels in the image, the egg dimensions in millimeters can be calculated.

The dimensions of the eggs measured in this paper correspond to^17^ and are shown in the Fig. 5. The dimensions included the length of the egg from the anterior end to the posterior end (length of the green square), the widest part of the egg (width of the green box), the widest part at the anterior 1/3 end (width of the blue box) and the widest part at the 1/3 posterior end (width of the red box), and the ratio between egg length and width. A t-test with a significance level ($\alpha$) of 5% was performed to see the significance of the differences in the dimensions of *Ae. aegypti* and *Ae. albopictus*. The measurement and statistical test were conducted to compare and confirm the proposed method with morphometrics results of previous studies^14, 16–19^.

### Elliptic fourier analysis

Elliptic Fourier Analysis (EFA) works by representing the egg contour into a chain code $C = h_{1}h_{2}h_{3} \ldots h_{k}$, where *k* is the number of egg contour points. When each contour point has been encoded, the chain code is repeated so that it can be approximated by a Fourier series. The Fourier series representation of the contours for the *x* and *y* projections is defined as^39^

2 $$\begin{aligned} x(t)= & {} A_{0} + \sum _{n=1}^{\infty } a_{n} cos \frac{2 \pi nt}{T} + b_{n} sin \frac{2 \pi nt}{T} \end{aligned}$$

3 $$\begin{aligned} y(t)= & {} C_{0} + \sum _{n=1}^{\infty } c_{n} cos \frac{2 \pi nt}{T} + d_{n} sin \frac{2 \pi nt}{T} \end{aligned}$$

where $A_{0}$ and $C_{0}$ are the DC components of the Fourier series and n is the number of harmonics used to produce contour approximations. *T* is the period of the chain code or the time required to traverse all contour points. The Fourier coefficients $a_{n}$, $b_{n}$, $c_{n}$, and $d_{n}$ for the *x* and *y* projections are then defined by following^39^

4 $$\begin{aligned} a_{n}= & {} \frac{T}{2 \pi ^{2} n^{2}} \sum _{p=1}^{K} \frac{\Delta x_{p}}{\Delta t_{p}} \left[ cos \frac{2 \pi n t_{p}}{T} - cos \frac{2 \pi n t_{p-1}}{T} \right] \end{aligned}$$

5 $$\begin{aligned} b_{n}= & {} \frac{T}{2 \pi ^{2} n^{2}} \sum _{p=1}^{K} \frac{\Delta x_{p}}{\Delta t_{p}} \left[ sin \frac{2 \pi n t_{p}}{T} - sin \frac{2 \pi n t_{p-1}}{T} \right] \end{aligned}$$

6 $$\begin{aligned} c_{n}= & {} \frac{T}{2 \pi ^{2} n^{2}} \sum _{p=1}^{K} \frac{\Delta y_{p}}{\Delta t_{p}} \left[ cos \frac{2 \pi n t_{p}}{T} - cos \frac{2 \pi n t_{p-1}}{T} \right] \end{aligned}$$

7 $$\begin{aligned} d_{n}= & {} \frac{T}{2 \pi ^{2} n^{2}} \sum _{p=1}^{K} \frac{\Delta y_{p}}{\Delta t_{p}} \left[ sin \frac{2 \pi n t_{p}}{T} - sin \frac{2 \pi n t_{p-1}}{T} \right] \end{aligned}$$

where $\Delta t_{p}$ is the time required to traverse the link p of the two contour points. $\Delta x_{p}$ and $\Delta y_{p}$ are the spatial changes in the *x* and *y* projections of the *p*-th link of the chain code. Each harmonic has 4 coefficients $a_{n}$, $b_{n}$, $c_{n}$, and $d_{n}$, yielding $4 \times n$ Elliptic Fourier (EF) coefficients. Referring to^39^, 3 EF coefficients of the first harmonic are used to normalize the overall EF coefficients, so that the number of normalized EF coefficients is reduced to $(4 \times n)-3$. This normalization process makes the EF coefficient to be invariant to the rotation, size, and starting point of the contour. Table 1 shows the example of normalized EF coefficients of one egg contour. EFD feature extraction in this study was carried out using pyEFD library^40^.

**Table 1:** Example of normalized EF coefficients of egg contour.

Harmonic	$a_{n}$	$b_{n}$	$c_{n}$	$d_{n}$
1	1.000000	− 2.7E−17	− 7.5E−17	− 0.3645
2	0.002165	− 0.00081	− 0.00161	0.021941
3	0.097114	0.000348	− 0.00704	− 0.0431
4	0.002283	0.001855	− 0.00417	0.00468
5	0.031059	0.000585	− 0.00035	− 0.01095
6	0.003075	0.000142	− 0.00222	0.001503
7	0.014326	0.000958	− 0.00163	− 0.00712
8	0.002306	0.001175	− 0.0005	− 0.00151
9	0.007903	0.001297	0.000414	− 0.00373
10	− 3.1E−05	0.001209	− 0.00144	− 0.00193

Furthermore, Principal Component Analysis (PCA) was carried out on the EF coefficients data set. PCA is widely used to reduce the dimensions of a data set that has a large number of variables and is correlated with each other while maintaining as much variance as possible in the data set. This can be done by reexpressing the data set into a new subspace using a transformation matrix called the principal component (PC). The resulting new variables or PCs have been sorted so that the first few PCs contain most of the variance of all the original variables. Since PCA in this study is used to reduce dimensions, so that an analysis is needed to select the number of PCs to be used. The analysis on the selection of the number of PCs was carried out based on statistical tests and *Wilk’s Lambda*^41^.

### Classification

The use of the Linear Discriminant Analysis (LDA) algorithm in this study was based on previous studies^30, 31, 35, 36^. The classification process began by randomly selecting 20 samples of each species from the data set so that as many as 40 samples of test data are obtained that will be used to evaluate the performance of the model. The remainder of the data set was then used to train the model with a total of 48 and 52 samples for *Ae. aegypti* and *Ae. albopictus*, respectively. *Ae. aegypti* eggs are labeled by 0 and *Ae. albopictus* eggs are labeled by 1.

**Table 2:** Confusion matrix for model evaluation.

		Predicted	
		*Ae. aegypti*	*Ae. albopictus*
Actual	*Ae. aegypti*	TN	FP
	*Ae. albopictus*	FN	TP

**Table 3:** Alternative algorithms and hyperparameter configuration.

Alternative	Hyperparameter	Types	Search
Algorithms			Space
LDA	solver	Categorical	[’lsqr’, ’eigen’]
	shrinkage	Continuous	[0, 1]
RF	n_estimator	Discrete	[10, 100]
	max_depth	Discrete	[5, 50]
	min_samples_split	Discrete	[2, 11]
	min_samples_leaf	Discrete	[1, 11]
	criterion	Categorical	[’gini’, ’entropy’]
	max_features	Discrete	[1, 64]
SVM	C	Continuous	[0.1, 50]
	kernel	Categorical	[’linear’, ’poly’, ’rbf’, ’sigmoid’]
KNN	n_neighbords	Discrete	[1, 20]

In its place, common machine learning algorithms such as Random Forest (RF), Support Vector Machine (SVM), and *k*-Nearest Neighbors (KNN) were also used. Alternative algorithm training was carried out with hyperparameter tuning to get the optimal model using Random Search with *k*-fold cross-validation^42^. The *k* value used for cross-validation is 10. The selection of alternative algorithms and hyperparameter configurations for tuning at this stage is carried out based on^43^ as shown in Table 3. Furthermore, to evaluate each model, a confusion matrix is used as shown in Table 2, where *TN*, *TP*, *FP*, and *FN* are True Negative, True Positive, False Positive, and False Negative, respectively. Performance metrics used in this study are accuracy and F1-score which can be calculated by the following equation

8 $$\begin{aligned} Accuracy= & {} \frac{TP+TN}{TP+FP+FN+TN} \end{aligned}$$

9 $$\begin{aligned} Precision= & {} \frac{TP}{TP+FP} \end{aligned}$$

10 $$\begin{aligned} Recall= & {} \frac{TP}{TP+FN} \end{aligned}$$

11 $$\begin{aligned} F1-score= & {} 2 \times \frac{Recall \times Precision}{Recall + Precision} \end{aligned}$$

## Results and discussions

### Egg’s dimensions

Based on the dimension measurements and t-test carried out in this study, *Ae. aegypti* eggs were significantly longer than *Ae. albopictus*’ (*p* value $< 0.000$), similar with results reported by^16–18^. In this study, *Ae. aegypti* eggs were also significantly wider (*p* value $< 0.000$) similar to^17^, but not narrower as reported by^16^. The widths at the anterior 1/3 end and 1/3 posterior end of the two species’ eggs were also significantly different (*p* value $< 0.000$), which is also similar to that reported by^17^. However, the ratio of length and width was not significantly different (*p* value = 0.542) as the results obtained in^17^. All the measurement results are in mean ± standard deviation millimeters as shown in the Table 4. This measurement process was carried out using *OpenCV* library and t-test using stats library in *Python 3.7.3*.

The developed method results in the ratio of the mean of egg length and width 1.0057 compared to 1.0118 in^17^. The difference of the new result is 0.6028% to the previous result. The difference is very small, it indicates that the image-based method for dimension identification is comparable with the result using SEM.

**Table 4:** Comparison of *Ae. aegypti* and *Ae. albopictus* eggs’ dimensions.

Attributes	*Ae. aegypti*	*Ae. albopictus*	*p* value
Egg length	$0.584 \pm 0.030$	$0.516 \pm 0.020$	< 0.000
Egg width	$0.159 \pm 0.009$	$0.141 \pm 0.006$	< 0.000
Egg width at 1/3 ant. end	$0.155 \pm 0.009$	$0.138 \pm 0.006$	< 0.000
Egg width at 1/3 post. end	$0.141 \pm 0.008$	$0.121 \pm 0.006$	< 0.000
Egg length and width ratio	$3.686 \pm 0.234$	$3.665 \pm 0.164$	0.542

**Figure 6:**
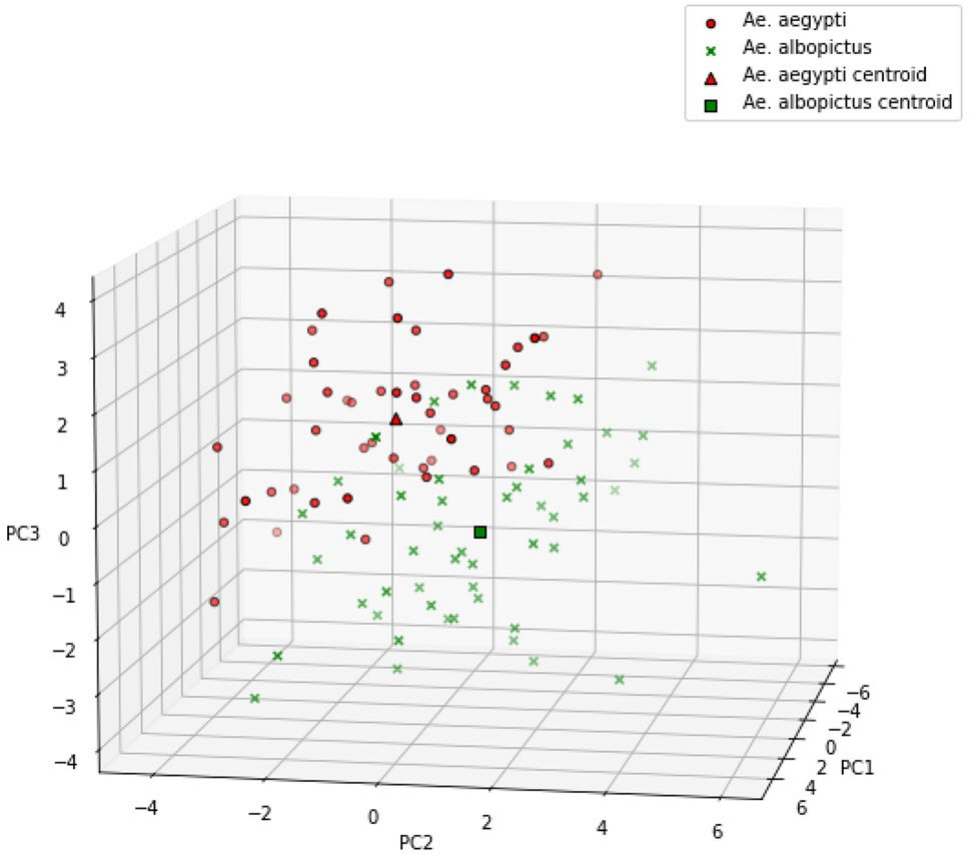
*Ae. aegypti* and *Ae. albopictus* data points in PC feature space.

### Dimensionality reduction

The analysis began with performing PCA to the EF coefficient data set. PCA transforms the EF coefficient into a feature space with 37 new uncorrelated variables called PC1 to PC37. Based on the PC values, a statistical analysis called Multivariate Analysis of Variance (MANOVA) was performed to see how significant the difference between the two types of eggs was. MANOVA is a hypothesis testing method that compares the average data for each class that has 2 or more variables. The statistical test conducted in this study was carried out using IBM SPSS Statistic v.26 software with a significance level ($\alpha$) of 5%.

Before MANOVA can be performed, there are ideal assumptions that must be met. Namely, each variable in each class must be normally distributed (normality) and the population covariance matrices must be equal (homogeneity).

**Table 5:** MANOVA results of *n* first PCs.

*n* first PC	Wilk’s lambda	*p* value	$H_{0}$ decision
2	0.716	$< 0.000$	Rejected
3	0.411	$< 0.000$	Rejected
4	0.411	$< 0.000$	Rejected

From the results of the *Kolmogorov-Smirnov* normality test, it is known that all PCs are normally distributed, except PC11 and PC37. Therefore, it can be concluded that all PCs meet the assumption of normality except PC11 and PC37. In the homogeneity test using *Box’s M*, it is known that the assumption of homogeneity is only met when the first 2 PCs, the first 3 PCs, or the first 4 PCs are used. Based on the two ideal assumption tests, the MANOVA test was only carried out using only the first 2, 3, and 4 PCs. Table 5 displays the results of the MANOVA test on n first PCs. The results yielded *p* value $< 0.000$ for all options. This indicates that there is a significant difference in the two types of eggs based on the PC value. In^41^, it is stated that the WL value has an effect on misclassification and can be used as a feature selection method. In general, the smaller the WL value is associated with the better classification results. By considering WL value from the MANOVA test results, in this study the first 3 PCs were used because the first 4 PCs did not get a smaller WL value. By using the first 3 PCs,  a visualization of data distribution of *Ae. aegypti* and *Ae. albopictus* eggs in PC feature space can be generated as shown in the Fig. 6. Data points distribution of two vectors’ eggs are overlapping at a certain region due to the shape similarity of both eggs.

**Figure 7:**
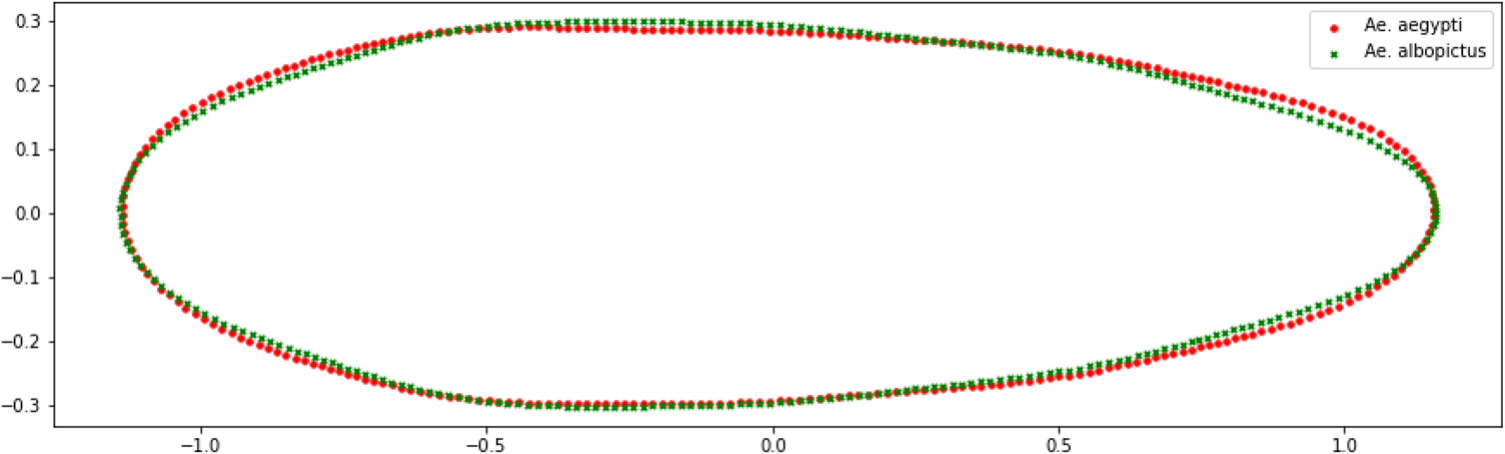
Comparison of average *Ae. aegypti* and *Ae. albopictus* egg contours.

**Figure 8:**
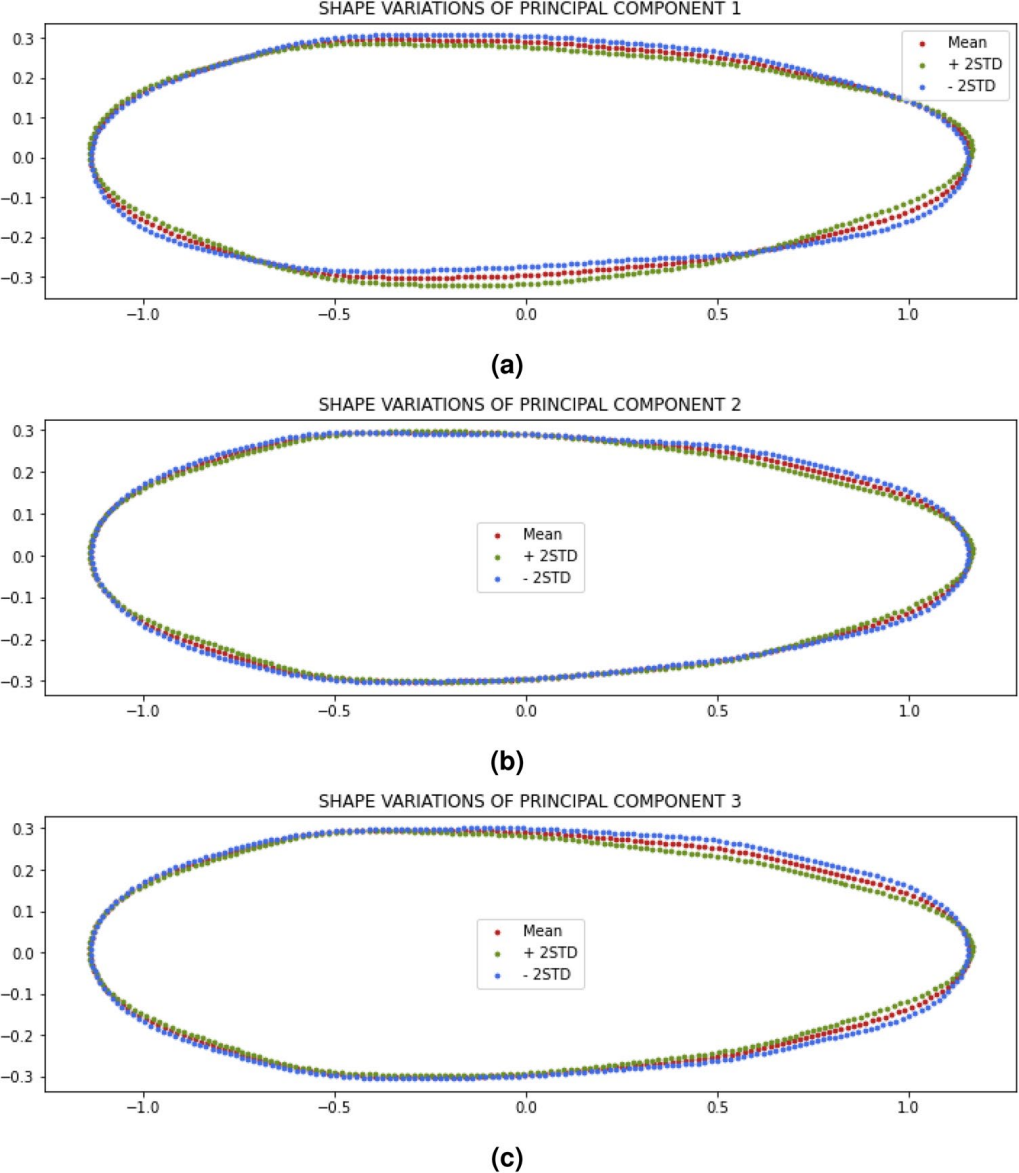
(**a**) Shape variation of PC1, (**b**) shape variation of PC2, and (**c**) shape variation of PC3.

### Egg’s shape contour analysis

Reconstruction of the average contour is done by calculating the average value of the EF coefficient of each class and visualization is obtained as shown in Fig. 7. Normalization of the EF coefficient of EFD produces a contour with the position of the anterior end of the egg on the left side and the posterior end of the egg on the right side. Normalization of the EF coefficient also makes the contours have the same length so that variations in differences can only be observed in the direction of the *y*-axis or egg width.

The average contour of the eggs of *Ae. aegypti* is generally cigar-shaped with the widest part at the 1/3 anterior end, tends to be straight at 1/3 middle, and tapered at 1/3 posterior end. The average contour of the eggs of *Ae. albopictus* is generally cigar-shaped with the widest part at 1/3 anterior end and tapering directly from 1/3 middle towards the posterior end. This description of egg shape is similar to that described by^16^ regarding the general shape of the eggs of *Ae. aegypti* and *Ae. albopictus*.

To be able to find out the location of the variations in the shape, contour reconstruction was carried out based on each PC. This process was done by performing an inverse transform using the PC scores vector and certain eigenvectors so that the EF reconstruction coefficient values are obtained. From the EF coefficients, the average and standard deviation were calculated and the EFD inverse transform was carried out so the contour coordinates were recovered to be visualized. The results of the reconstruction of PC1, PC2, and PC3 are shown in Fig. 8.

As shown in Fig. 8, PC1 corresponds to the curved direction of the egg. The positive value of PC1 makes the egg contour slightly curved upwards, while the negative value makes the egg contour slightly curved downwards. PC2 corresponds to the shape of the egg at the anterior and posterior end. The values in the positive direction makes the width at the end of the egg wider. While the value in the negative direction makes the width smaller. PC3 corresponds to the shape of the egg at the anterior end and especially the middle to the posterior end. The values in the positive direction makes the egg more tapered at the middle to posterior end. While the value in the negative direction makes the middle to posterior less taper.

Based on the results of statistical analysis and contour reconstruction, the results are in accordance with previous studies which stated that *Ae. albopictus* eggs were more tapered at the posterior end^16–18^.

**Figure 9:**
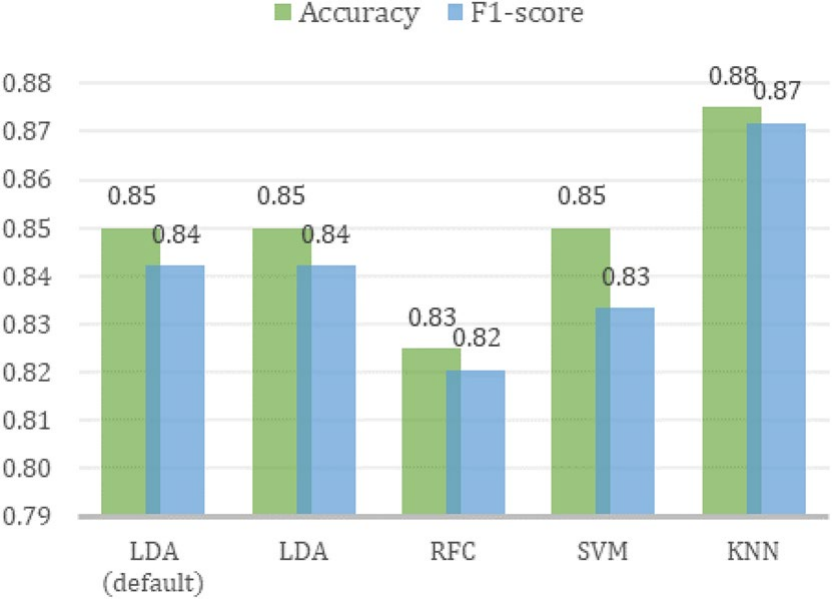
Comparison of optimal models’ performance on test data.

**Figure 10:**
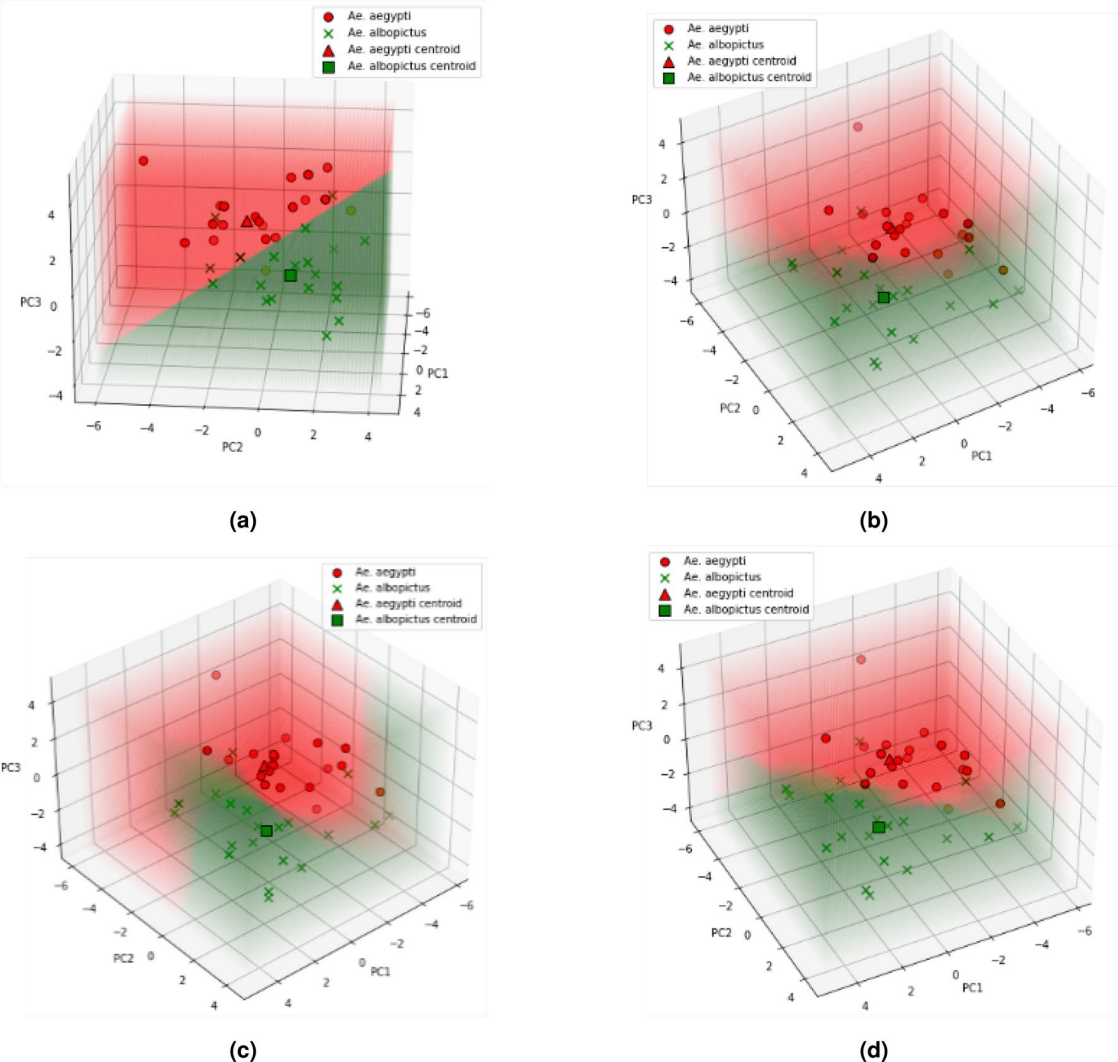
Test data in decision boundaries of optimal (**a**) LDA, (**b**) RF, (**c**) SVM, and (**d**) KNN model.

### Classification

LDA classification with default hyperparameters was used as a baseline for the comparison of model performance. The classification evaluation process at this stage was carried out by training the LDA model using train data and then evaluating it with test data. The performance of the baseline model on the test data is shown in the Table 6. By using Eqs. (8), (9), (10), and (11) the accuracy and $F_{1}$ score could be calculated as 0.85 and 0.84, respectively.

**Table 6:** LDA default confusion matrix on test data.

		Predicted	
		*Ae. aegypti*	*Ae. albopictus*
Actual	*Ae. aegypti*	**18**	2
	*Ae. albopictus*	4	**16**

Significant values are in bold.

The hyperparameter tuning of LDA and classification using alternative algorithms and *k*-fold cross-validation as described in “Classification” section yielded classification results as shown in Fig. 10. The hyperparameter tuning of the LDA algorithm did not yield better performance. As shown in Fig. 7, the data distribution of *Ae. aegypti* and *Ae. albopictus* in the PC feature space is not completely linearly separated due to similarity of the two species’ eggs. Since the LDA algorithm classifies the data using a linear equation, poor performance is obtained on class data that are not linearly separated in their feature space. Linearity itself is one of the shortcomings of LDA^44^.

The decision boundary generated from the training data of each algorithm is shown in the Fig. 10. The red region is *Ae. aegypti* class area and the green region is *Ae. albopictus* class area in the PC feature space. LDA produces a linear hyperplane while RF, SVM, and KNN produce a nonlinear hyperplane. Although the three alternative algorithms are all nonlinear classifiers, the best classification performance was obtained using the KNN algorithm with an accuracy of 0.88 and an $F_{1}$score of 0.87 on the test data. This result shows promising results for initial research of automatic identification of *Ae. aegypti* and *Ae. albopictus* eggs, since there are currently no studies regarding these two species’ eggs classification. It also implies that the use of nonlinear classifier was able to improve the classification performance of this method, instead of only using LDA^30, 31, 35, 36^.

Compared to SVM, KNN is known to have better performance when the amount of training data is more than the number of variables or features. Whereas SVM generally works well on datasets with many features^45^. On the other hand, although RF works well with small datasets, it tends to be very sensitive to changes in datasets and the number of features. It makes RF easily overfit and affects the classification performance^46^. The classification process in this study used 100 egg data with a much smaller number of features, namely the values of PC1, PC2, and PC3. This nature of the dataset made KNN outperforms the other classifier algorithms and yields a higher classification result.

### Limitations and further developments

The proposed method yielded promising results for initial research on the automatic identification of *Ae. aegypti* and *Ae. albopictus*’ eggs. However, there are several limitations and further developments regarding this study which we will discuss in this section.

The main limitation of this study is the limited species of interest. This study only focused on *Ae. aegypti* and *Ae. albopictus* which will not be applicable beyond the analyzed species. Additional species are essential for further studies, since there may be a percentage of other species in real-field ovitrap-based settings. Egg’s condition classification will also be very helpful in this context, for example by adding a classification model to differentiate between egg objects and non-egg objects before the species identification process.

This study was also conducted in a laboratory-conditioned setting, where the eggs are selected, observed, captured, and validated individually. This leads to a non-practical application of the proposed methods since there are a lot of variety in real-field settings. Fortunately, we covered the variety of egg sizes where the egg contour was normalized as discussed in the previous section.

Other image processing methods will also be very important for further development to overcome the non-practical limitation of this study. One possible improvement is by adding an overlapping-object separation method in the image processing stage. This is due to the real condition in ovitraps-collected eggs in which they are often found to be clustered and overlap each other. Moreover, using a higher-resolution camera will also be very advantageous for further studies.

## Conclusions

This paper proposed the method to automatically identify *Ae. aegypti* and *Ae. albopictus* eggs based on digital images using a smartphone. From all the results, the proposed method confirmed that the egg dimensions of *Ae. aegypti* and *Ae. albopictus* and the shape at the posterior end differs significantly (*p* value $< 0.000$) similar to as mentioned in previous studies. The classification based on first 3 PC using KNN yielded an accuracy and F1-score of 0.88 and 0.87, respectively. This provides the possibility of automatic egg identification based on digital images.

By combining the proposed method with an overlapping-objects separation method at the image processing stage, the real implementation can be done with few adjustments. This can help epidemiologists to identify the two eggs more efficiently without having to carry out observations in a time-consuming conventional way. This digital image-based automatic identification can also assist authorized officials in monitoring and making decisions in developing vector control strategies.
